# The Sertoli Cell Complement Signature: A Suspected Mechanism in Xenograft Survival

**DOI:** 10.3390/ijms24031890

**Published:** 2023-01-18

**Authors:** Rachel L. Washburn, Dalia Martinez-Marin, Ksenija Korać, Tyler Sniegowski, Alexis R. Rodriguez, Beverly S. Chilton, Taylor Hibler, Kevin Pruitt, Yangzom D. Bhutia, Jannette M. Dufour

**Affiliations:** 1 Department of Cell Biology and Biochemistry, School of Medicine, Texas Tech University Health Sciences Center, Lubbock, TX 79424, USA; 2 Department of Immunology and Molecular Microbiology, School of Medicine, Texas Tech University Health Sciences Center, Lubbock, TX 79404, USA

**Keywords:** Sertoli cells, immune regulation, complement, transplantation, xenografts

## Abstract

The complement system is an important component of transplant rejection. Sertoli cells, an immune regulatory testicular cell, survive long-term when transplanted across immunological barriers; thus, understanding the mechanisms behind this unique survival would be of great benefit to the transplantation field. This study focused on Sertoli cell inhibition of complement as relevant in xenotransplantation. Neonatal pig Sertoli cells (NPSCs) survived activated human complement in vitro while neonatal pig islet (NPI) aggregates and pig aortic endothelial cell (PAEC) survival were diminished to about 65% and 12%, respectively. PAECs cultured in NPSC-conditioned media and human complement demonstrated a 200% increase in survival suggesting that NPSCs secrete complement-inhibiting substances that confer protection. Bioinformatic and molecular analyses identified 21 complement inhibitors expressed by NPSCs with several significantly increased in NPSCs compared to NPIs or PAECs. Lastly, RNA sequencing revealed that NPSCs express 25 other complement factors including cascade components and receptors. Overall, this study identified the most comprehensive Sertoli cell complement signature to date and indicates that the expression of a variety of complement inhibitors ensures a proper regulation of complement through redundant inhibition points. Understanding the regulation of the complement system should be further investigated for extending xenograft viability.

## 1. Introduction

Sertoli cells are testicular cells with immunoregulatory properties. Within the scope of male reproduction, Sertoli cells function to nurture the maturing germ cells, encourage proper progression of germ cells through spermatogenesis, and protect germ cells from autoimmune destruction [1]. These immunoregulatory properties led to transplantation studies where it was found that Sertoli cells survive as both allografts and xenografts long-term, >90 days, without immune suppressants [2], which is quite remarkable since grafts—especially xenografts—require extensive immunosuppressant therapy to fend off rejection by the recipient’s immune system [3]. This unique quality of Sertoli cells makes them an attractive model for studying immunoregulatory mechanisms that will benefit the fields of transplantation, chronic inflammatory disease, and autoimmune disease. This study focuses on the Sertoli cell immune regulation of the complement system.

**Figure 1:**
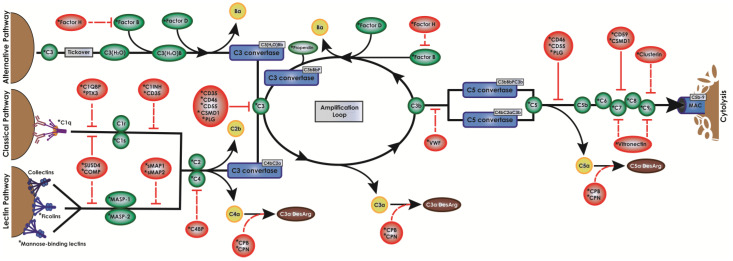
The complement cascade. Complement is a cytolytic cascade that can be activated spontaneously (alternative pathway), by antibody binding (classical pathway), or by bacterial oligosaccharides (lectin pathway). All three pathways converge on the creation of the C3 and C5 convertases, which lead to the assembly of the MAC. Red bubbles indicate complement inhibitors. Green bubbles indicate complement cascade components. Blue bubbles indicate enzymatic convertase complexes. Yellow bubbles indicate anaphylatoxins. Brown bubbles indicate des-arginated forms of anaphylatoxins. * indicate complement genes expressed by NPSCs.

A major component of the immune response in transplant rejection is the complement system [4]. Complement is an important, complex, and often unappreciated proteolytic cascade that is one of the first responders against invading microbes and pathogens (Figure 1). When complement is activated upon xenograft introduction, the grafted tissue is almost always destroyed [4]. Once activated by either antibody-antigen complexes (classical pathway), microbial lectins (lectin pathway), or spontaneously (alternative pathway), complement forms two major convertases (C3 and C5 convertases) that cleave the components C3 and C5. These cleavage products can opsonize the cell to encourage phagocytosis, are released into the surrounding tissue and circulation where they recruit and activate immune cells to aid in the antimicrobial response, and/or contribute to the assembly of the cytolytic membrane attack complex (MAC). As uncontrolled complement activation leads to massive collateral damage, host cells express complement inhibitors to protect themselves (Figure 1, red bubbles). These inhibitors act as kill switches to all major parts of the complement system, preventing cell lysis and uncontrolled inflammation.

Previously, we have shown that neonatal pig Sertoli cells (NPSCs) exposed to human serum and rabbit complement in vitro survived at over 100% whereas neonatal pig islet (NPI) controls and pig aortic endothelial cell (PAEC) controls survived at under 40% and 30%, respectively [5]. Furthermore, MAC deposition was not detected with immunohistochemical analysis of NPSCs exposed to activated rabbit complement in vitro or NPSCs grafted under the kidney capsule of xenogeneic rats in vivo, while controls (PAECs in vitro or NPIs in vivo) were positive for MAC [5,6]. These studies indicate that xenogeneic Sertoli cells are inhibiting the complement system before MAC insertion. A mechanism they may utilize to regulate complement is to express complement inhibitors. Hence, we hypothesize that pig Sertoli cells express a unique complement signature that contributes to their survival as xenografts.

Therefore, in this study we conducted a clinically relevant in vitro human complement serum (HCS, human serum with complement and antibodies preserved) cytotoxicity assay to measure the survival of NPSCs after exposure to activated human complement. Next, we performed RNA sequencing on our transplantable population of NPSCs and used bioinformatic analysis along with qPCR and ELISA to establish a baseline expression of complement inhibitory proteins. Then, we compared the expression of the complement inhibitory proteins against the NPI and PAEC controls. To determine whether NPSCs secrete complement inhibitors that are protective in a robust human complement environment, PAECs were cultured in Sertoli cell-conditioned media (SCCM) and then exposed to the HCS cytotoxicity assay where their survival doubled. Lastly, we analyzed the RNA sequencing data for the NPSC expression of other major complement factors including cascade components and receptors, then compared the expression for significant differences between NPSCs and NPIs. NPSCs expressed mRNA for 25 other complement factors that may be involved in other immunomodulatory processes.

## 2. Results

### 2.1. Pig Sertoli Cells Survive Human Complement

**Figure 2:**
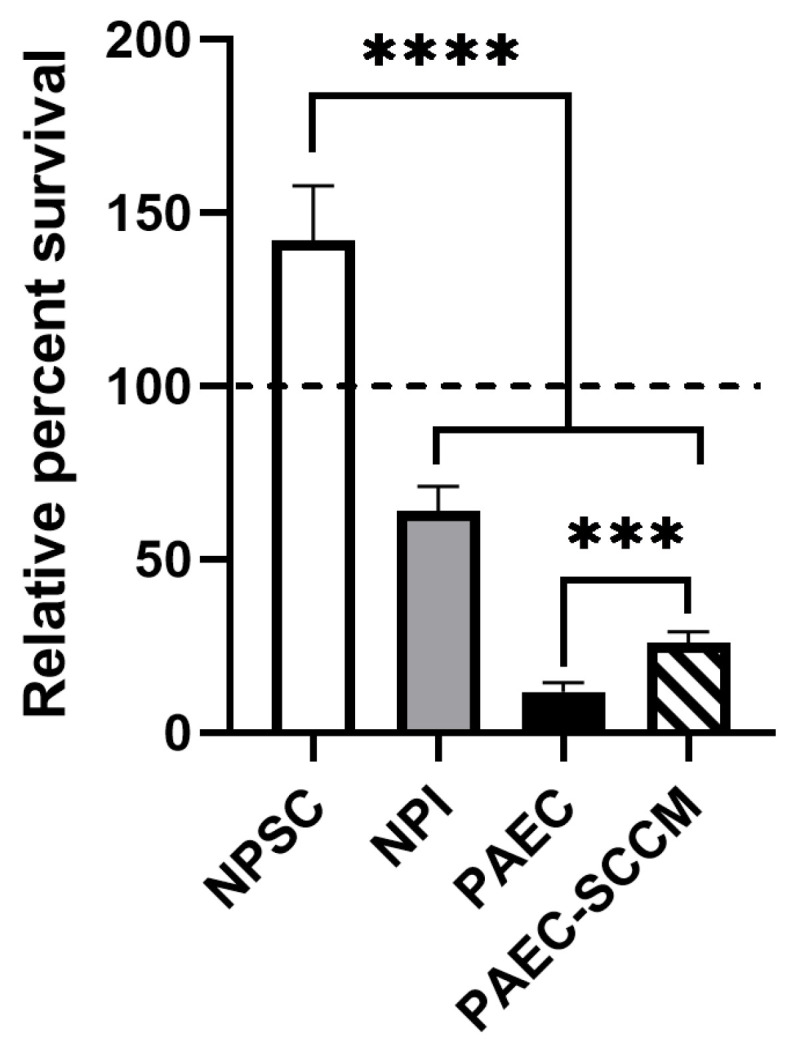
NPSC survival in human complement in vitro. NPSCs (white bar) survive at 141.8 ± 6.5% after exposure to activated human complement serum for 90 min while NPI (gray bar) and PAEC (black bar) controls are diminished to 64 ± 4.0% and 11.7 ± 1.4%, respectively. PAECs cultured in media collected from NPSCs (PAEC-SCCM, striped bar) survive human complement in vitro at about 25.9 ± 1.6%. Results were normalized to media-only controls of 100% (dashed line). Statistical significance was determined with unpaired t-test with two-tailed *p*-value. *** *p* < 0.001. **** *p* < 0.0001.

NPSCs and control cells (NPIs and PAECs) were cultured in HCS for 1.5 h, then assayed for survival. Results were normalized to media controls. NPSC relative percent survival averaged 141.8 ± 6.5%, while NPI survival was 64 ± 4.0% and PAEC survival was 11.7 ± 1.4% (Figure 2).

### 2.2. Pig SCCM Significantly Increases PAEC Survival of Human Complement

To determine whether NPSCs secrete factors that inhibit complement-mediate killing, we collected SCCM from NPSCs for use in the HCS cytotoxicity assay. PAECs were cultured for 1.5 h in either media and HCS (1:1) or SCCM and HCS (1:1), then assayed for survival. Results were normalized to media-only controls (Figure 2). PAECs cultured in SCCM with HCS exhibited significantly elevated survival at 25.9 ± 1.6% compared to PAECs in normal media and HCS, which survived at under 12% (Figure 2).

### 2.3. RNA Sequencing Analysis Identified That Pig Sertoli Cells Express Genes for at Least 22 Complement Cascade Components and 3 Complement Receptors

**Figure 3:**
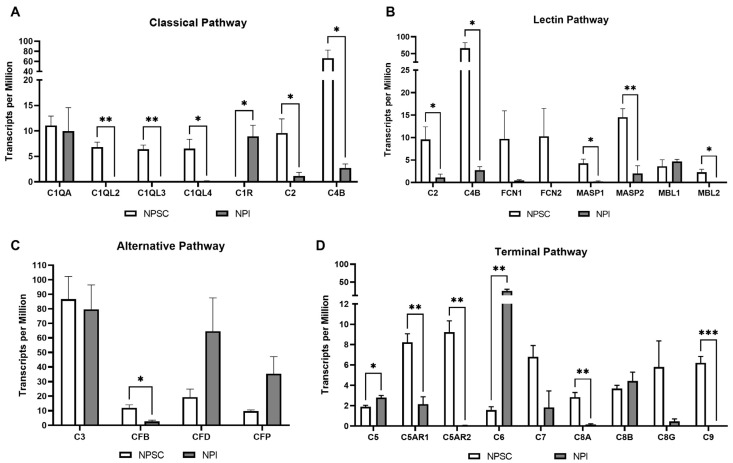
Complement component expression by NPSCs versus NPIs. RNA sequencing of classical pathway (**A**), lectin pathway (**B**), alternative pathway (**C**), and terminal pathway (**D**) complement components expressed by NPSCs (white bars) and NPIs (gray bars). Statistical significance was determined with the unpaired *t*-test. Note: *C3* is included in the alternative pathway but also serves as a convergence point for all three activation pathways as indicated in Figure 1. * *p* ≤ 0.05. ** *p* ≤ 0.01. *** *p* ≤ 0.001.

Of interest, RNA sequencing analyses also identified gene expression of 25 other complement factors/receptors by NPSCs (Figure 3, Table S1). These include the classical pathway component genes C1q (C1qA, C1qL2, C1qL3, C1qL4), C2, and C4B (Figure 3A); lectin pathway component genes (Figure 3B) ficolin-1 (FCN1), ficolin-2 (FCN2), mannose-binding lectin-1 (MBL1), MBL2, MBL-associated serine proteinase-1 (MASP1), and MASP2; the alternative pathway component genes including C3, complement factor B (CFB), complement factor D (CFD), and complement factor properdin (CFP) (Figure 3C); the convertase components including C3 and C5; the terminal pathway component genes C6, C7, C8α, C8β, C8γ, and C9 (Figure 3D); and the receptor genes C5aR1 and C5aR2 (Figure 3D). NPSCs have significantly elevated gene expression of the complement factors C1q, C2, C4B, CFB, C5aR1, C5aR2, C8α, C9, MASP1, MASP2, and MBL2 as compared to NPIs. NPSCs also demonstrated significantly decreased gene expression of the complement components C1r, C5, and C6. NPSCs express the complement component genes C3 (86.65 ± 13.51 TPM) and C4B (66.54 ± 13.90 TPM) at the highest levels as compared to the other components/receptor genes and have the least gene expression of C1r (0 TPM). As the pig (sus scrofa) genome has not yet been fully annotated, genes encoding for any complement components not mentioned (i.e., C1s, C3aR, C4A, etc.) may be expressed by NPSCs, but this is not yet verified.

### 2.4. RNA Sequencing Identified Pig Sertoli Cells Express mRNA for 21 Complement Inhibitors

**Figure 4:**
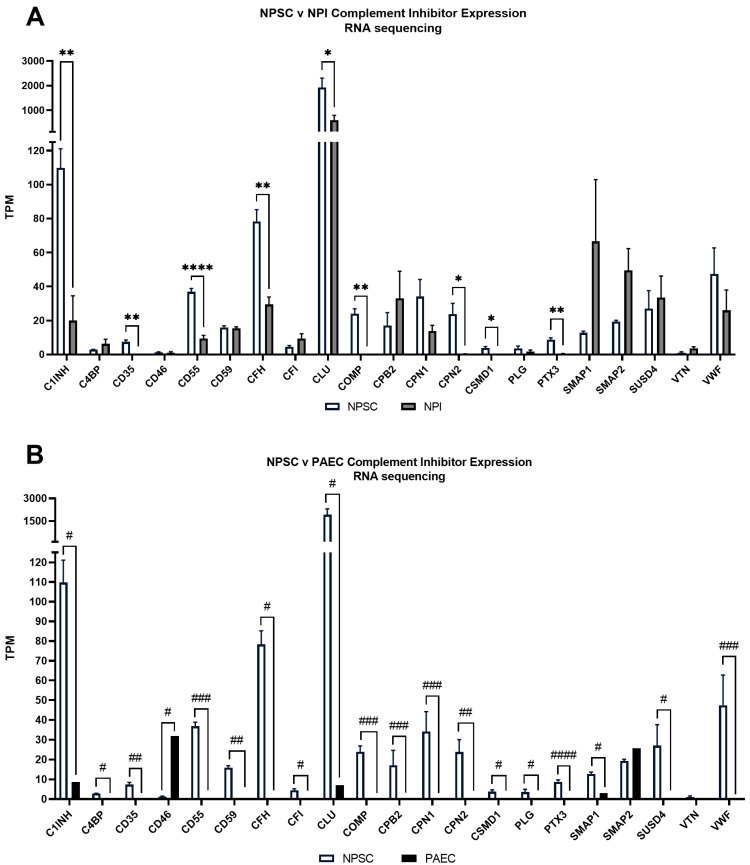
NPSCs express 21 complement inhibitor genes. (**A**) RNA sequencing identified NPSCs (white bars) express nine complement inhibitor genes at significantly elevated levels compared to NPIs (gray bars). Statistical significance was determined with unpaired *t*-test. Numerical values are contained in Table S2. Asterisks denote significance. * *p* ≤ 0.05. ** *p* ≤ 0.01. **** *p* < 0.0001. (**B**) Mean comparison of RNA sequencing for complement inhibitor genes between NPSCs (white bars) and PAECs (black bars). Log_2_ Fold Change (LFC) was calculated between Sertoli cells and endothelial cells for comparison of data sets. Numerical values are contained in Table S3. Pound sign denotes significance. # LFC > 1. ## LFC > 5. ### LFC > 10. #### LFC > 20.

RNA sequencing analyses revealed that NPSCs express 21 complement inhibitory proteins (Figure 3, Table 1): C1 inhibitor (C1INH), C4 binding protein (C4BP), CD35, CD46, CD55, CD59, complement factor H (CFH), complement factor I (CFI), clusterin (CLU), cartilage oligomeric matrix protein (COMP), carboxypeptidase B2 (CPB2), CPN1, CPN2, Cub and Sushi multiple domain protein 1 (CSMD1), plasminogen (PLG), pentraxin (PTX3), small MBL-associated protein-1 (SMAP1), SMAP2, sushi-domain containing protein 4 (SUSD4), vitronectin (VTN), and von Willebrand factor (VWF). The RNA sequencing data set was then compared against a previously published data set for NPIs (Figure 4A, Table S2) and analyzed for complement inhibitor expression. Nine of these inhibitors have significantly elevated levels of gene expression in NPSCs as compared to NPIs (Figure 4A)—C1INH, CD35, CD55, CFH, CLU, COMP, CPN2, CSMD1, and PTX3—which inhibit at every major point of the complement cascade (Figure 1, Table 1).

Furthermore, analysis of the average transcripts per million (TPM) in RNA sequencing data comparing NPSCs to PAECs (Figure 4B, Table S3) identified that 18 out of these 21 complement inhibitors have elevated gene expression in NPSCs (Figure 4B). The only inhibitor with increased gene expression in PAECs was CD46. PTX3 had the largest gene expression increase in LFC between NPSCs and PAECs.

### 2.5. PCR Confirms That Pig Sertoli Cells Have Elevated Gene Expression of Several Complement Inhibitors

**Figure 5:**
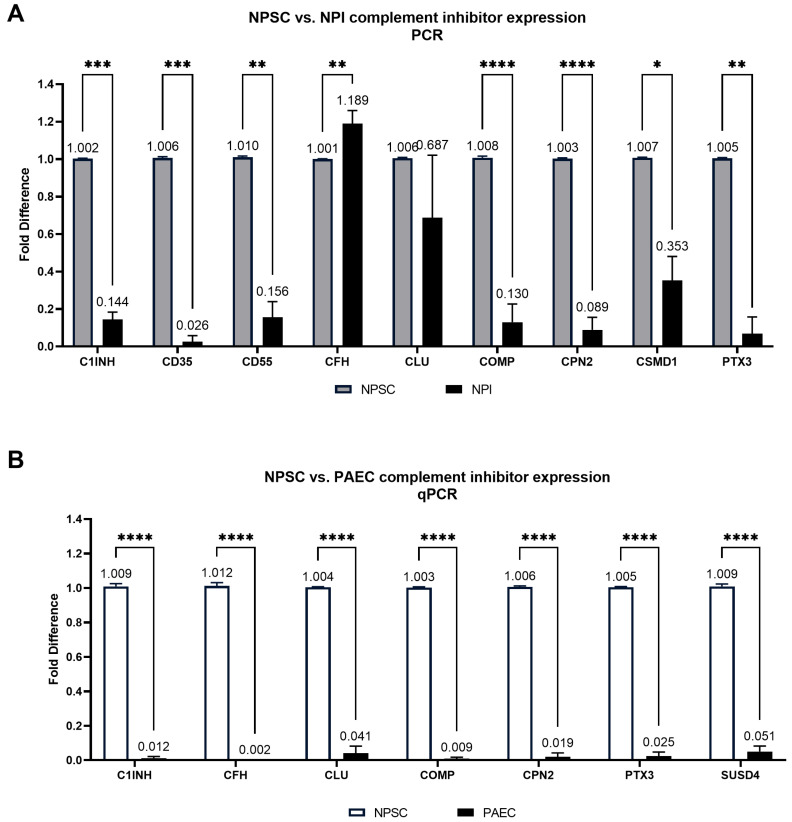
NPSCs have elevated gene expression of several complement inhibitors. (**A**) qPCR confirmed NPSCs (white bars) have elevated expression of seven complement inhibitor genes as compared to NPIs (gray bars). Numerical values are above each bar and are also contained in Table S5. (**B**) qPCR confirmed that NPSCs (white bars) have elevated expression of the seven soluble complement inhibitor genes as compared to PAECs (black bars). Numerical values are above each bar and are also contained in Table S6. Statistical significance was determined with unpaired t-test. Asterisks indicate significance. * *p* < 0.05. ** *p* < 0.01. *** *p* < 0.001. **** *p* < 0.0001.

Next, we performed qPCR on NPSCs and NPIs for the same nine inhibitors that were significantly elevated in NPSCs by RNA sequencing and confirmed that NPSCs express seven of the nine inhibitors at significantly elevated levels as compared to NPIs (Figure 5A, Tables S4 and S5). Gene expression of CLU was not significantly different between NPSCs and NPIs, and gene expression of CFH was elevated in NPIs. qPCR analyses of the seven soluble complement inhibitor genes between NPSCs and PAECs (Figure 5B, Tables S4 and S6) revealed that NPSCs have elevated gene expression of all seven: C1INH, CFH, CLU, COMP, CPN2, PTX3, and SUSD4 (Figure 5B).

### 2.6. Pig Sertoli Cells Secrete Complement Inhibtory Proteins

**Figure 6:**
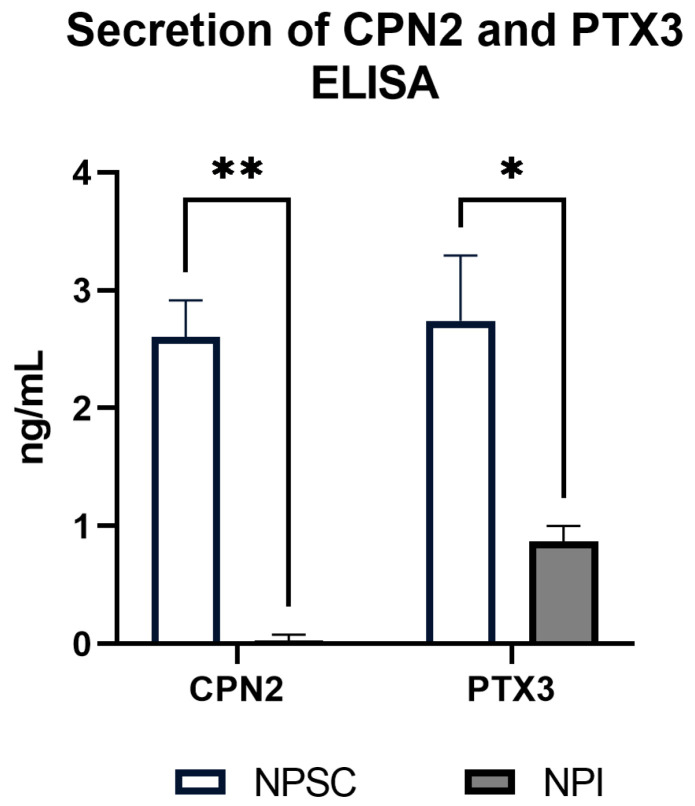
NPSCs and NPIs secreted protein expression of Cpn2 and Ptx3. ELISA assays were performed to quantify Cpn2 and Ptx3 protein secretion by NPSCs (white bar) and NPIs (gray bar). Significance was calculated using the unpaired t-test. * *p* < 0.05. ** *p* < 0.01.

Our lab has previously confirmed that mouse Sertoli cells express C1INH, CLU, and COMP protein [11] and that NPSCs express CD46, CD55, and CLU protein [6]. As SCCM is protective to an extent against complement, we decided to confirm and quantify NPSC protein expression of the secreted complement inhibitors Cpn2 and Ptx3 with an ELISA assay (Figure 6). CPN2 is an anaphylatoxin inhibitor that removes an arginine from C3a and C5a, forming C3adesArg and C5adesArg, respectively. C3adesArg cannot bind to the C3aR, which prevents inflammatory signaling through that pathway. C5adesArg has 10–15-fold less affinity than C5a to the C5aR1 receptor, reducing the inflammation-associated activity of this pathway [12,13]. NPSCs secrete Cpn2 at 2.60 ± 0.16 ng/mL, while NPI levels were significantly lower at 0.03 ± 0.02 ng/mL. PTX3 has a complicated role in various parts of the complement system, depending on which complement component is involved. For example, PTX3 interaction with activation components such as mannose-binding lectins or ficolins increases the repertoire of microbial factors recognized by complement [14]. Regarding its complement inhibitory function, PTX3 prevents activation of the classical pathway by inhibiting the C1 complex [15,16]. NPSCs secrete Ptx3 at levels of 2.74 ± 0.28 ng/mL, which is significantly elevated compared to NPI secretion of Ptx3 (0.87 ± 0.07 ng/mL).

## 3. Discussion

Since there have now been several clinical xenotransplantation studies, extending xenograft viability without increasing the immune suppressant requirement is becoming as important as extending allograft survival. The unique immunoregulatory nature of Sertoli cells may provide an answer to creating and maintaining a graft-accommodating environment. As Sertoli cells survive long-term without immunosuppressants when allografted and xenografted and have been shown to protect co-grafted cells [17,18], elucidating how they maintain an immune protective environment is imperative. The focus of this study was to identify the Sertoli cell complement signature by assessing the survival of pig Sertoli cells after incubation with human complement, their expression of complement inhibitory proteins, and their expression of other complement factors.

Previously, we have shown that NPSCs survive heat-inactivated human serum (inactive complement) with rabbit complement (active complement) while NPIs and PAECs have decreased survival to obly about 30–45% as compared to media-only controls [5]. In a more clinically relevant model, we confirmed that NPSCs had enhanced survival in human serum with human complement preserved in vitro. Under those same conditions, PAEC survival declined to under 12%, and NPI survival is decreased to around 65%. Interestingly, NPIs demonstrated increased survival to HCS compared to PAECs, which could be because the NPIs are cultured as aggregates of islet cells rather than a cell monolayer. The outer cells of the aggregates are more exposed to complement while the cells within the aggregates would be protected from complement. Another reason for this higher survival is that NPIs could also express complement inhibitors that provide them some protection from human complement. Indeed, we found that NPIs do express about a dozen complement inhibitors while PAECs express around five complement inhibitors.

RNA sequencing determined that NPSCs express complement cascade components, receptors, and inhibitors (Figure 3 and Figure 4). This is of particular interest as in the recent literature, intracellular complement (complosome) and local complement have been implicated as important signaling factors in cell survival, proliferation, and activation [19]. NPSCs express the genes for C3 and C4 at the highest levels, but this is not significantly different from NPI expression (Figure 3A,C). As the complosome seems to be important in normal cell physiology, expression of these two complement components may be a simple normal cell function [20]. Interestingly, the most significant differential expression of complement factors between NPSCs and NPIs was seen with ↑C1Q, ↓C1R, ↓C5, ↓C6, ↑C8A, ↑C9, ↑C5AR1, and ↑C5AR2 (Figure 3). Not much is known about C1r and C6 regarding transplant survival or immune regulation. The lower level of expression in NPSCs of these factors could indicate decreased classical pathway activation and MAC recruitment. C1q has recently been identified as an important part of the complosome in immune cell regulation, and the mechanism of its action is still under investigation [21,22]. Elevated gene expression of C1q in NPSCs could suggest a regulation of immune responses.

**Figure 7:**
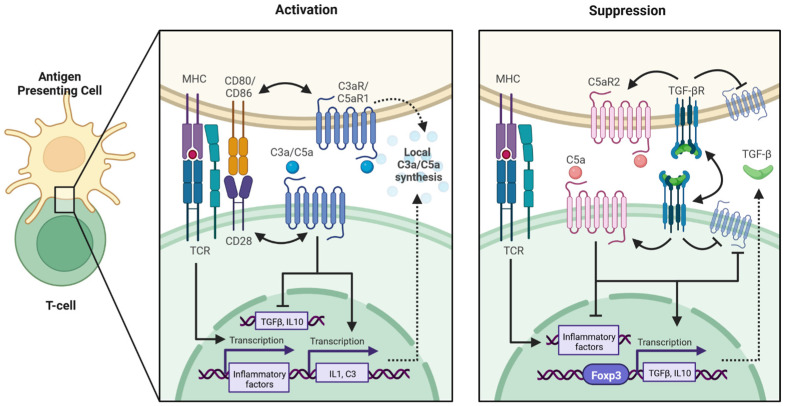
Complement anaphylatoxin signaling in T cell activation and suppression. Activation: Antigen presenting cells interact with T cells via MHC-TCR and CD80/86-CD28 complexes to activate signaling cascades that lead to expression of T cell inflammatory factors. Ligation of anaphylatoxin receptors C3aR and C5aR contribute to effector T cell activation by encouraging transcription of inflammatory cytokines such as IL1, inducing expression of C3, and by suppressing the immune suppressant factors TGF-β and IL-10. Suppression: When anaphylatoxin levels are diminished, C5aR2 sequesters C5a binding, inhibiting activation of the pro-inflammatory factors and allowing for expression of TGF-β and IL-10, which suppress effector immune response. This figure was created with Bio Render.

Very few cells have been shown to express so many complement cascade components, and these cells are primarily hepatocytes, renal cells, and immune cells (Table 2). Hepatocytes are the largest producers of most complement cascade components (except for C1q and C7) [23]. Macrophages, T cells, and B cells are the next highest producers of complement components, which allows them to mount an effective immune response [23,24,25]. Furthermore, the local production of complement—especially C3, C5, and their cleavage products—is absolutely required for antigen-presenting cells and T cells for Th1 and Th17 differentiation (Figure 7, Activation) [26,27,28]. There is also evidence that blocking C3aR and C5aR1 can lead to generation of immune suppressive regulatory T cells, which are associated with graft survival and the establishment of a graft-protective environment (Figure 7, Suppression) [29,30,31]. Interestingly, we have found that there are increased regulatory T cells in NPSC compared to NPI grafts [31]. Increased expression of C5aR2 by NPSCs could lead to decreased C5a levels and, thus, the generation of regulatory T cells. However, the elevated gene expression of C5aR1 and C5aR2 indicates the potential for NPSCs to respond to complement activation by usurping those pathways to modulate effector immune cell responses [19,28].

Given that NPIs xenografted into rats are rejected around day 4 post-transplantation and these xenografts are positive for the complement fragments C3 and MAC [6], complement is contributing to NPI destruction in vivo and in vitro. Both xenografted and allografted Sertoli cells survive these conditions, and MAC is not detected on Sertoli cell grafts [6,37]. One mechanism NPSCs may utilize to survive such a complement-rich environment is the expression of complement inhibitory proteins.

RNA sequencing analyses detected the gene expression for 21 different complement inhibitors by NPSCs, and 9 were significantly elevated as compared to NPIs (Figure 3A). Together, these complement inhibitors block all key points throughout the complement cascade (Figure 1, Table 1). CD35, CD46, CD55, CD59, CSMD1, and PLG are membrane-bound inhibitors that protect the expressing cell. C1INH, C1QBP, C4BP, COMP, CPB1, CPN1, CPN2, PLG, PTX3, sMAP1, sMAP2, SUSD4, CLU, VTN, and VWF are secreted proteins that have the potential to protect local cells from complement activation. C1INH, C1QBP, C4BP, CD35, COMP, CSMD1, PTX3, SMAP1, and SMAP2 all inhibit complement activation products. CD46, CD55, PLG, and VWF are inhibitors of the convertases which amplify complement. CD59, CLU, VTN are inhibitors of MAC and prevent the cytolytic portion of complement. CPB2, CPN1, and CPN2 inactivate anaphylatoxins, thus preventing inflammation, immune cell chemotaxis, and immune cell activation. Survival of PAEC exposed to HCS doubles when cultured in SCCM (Figure 2), suggesting the potential protective power of secreted complement inhibitors. It is feasible that increasing secreted complement inhibitor expression by knocking in inhibitor genes would increase PAEC survival to human complement. Future experiments should knock in the gene expression of some of these serum inhibitors, particularly PTX3 and the carboxypeptidases, into PAEC, then assess their survival to HCS—especially since the significance of secreted inhibitors has not yet been investigated in xenografts.

Regarding protein expression, we previously confirmed mouse Sertoli cell secretion of C1INH and COMP [11]. In this study, we confirmed NPSC secretion of CPN2 and PTX3 proteins, which was significantly increased as compared to NPIs (Figure 6). The formulation of a graft protective environment may require both types of complement inhibitors and inhibitors that can shut down excess complement activation at any step.

Some limitations to this study are that the pig genome has not been fully annotated, so it is likely that NPSCs could express more complement factors and inhibitors that may be identified in the future. Additionally, the availability of reagents for pig proteins such as western blot antibodies and ELISA kits is limited, so we were not able to quantify the complement inhibitor proteins aside from Cpn2 and Ptx3 at this time [6]. This limitation will hopefully be overcome in the future as more reagents become available for pig proteins, especially since xenotransplanted pig tissue is receiving increased attention in the field.

Overall, this study demonstrated that NPSCs survive human complement and that PAECs have increased survival to human complement when cultured in SCCM, and this study defined the most comprehensive Sertoli cell complement signature to date including complement inhibitors, complement factors, and complement receptors. As complement is being realized as an increasingly vital component of immune regulation and cellular physiology, the Sertoli cell complement signature may continue to provide insight into the potential of complement regulation in germ cell protection by testis immune privilege and in establishing a graft protective environment. Though this has a potential role in male reproduction and fertility, in transplantation this would allow for more widespread use of xenografted organs, increased xenograft and allograft viability, and decreased requirement for toxic immune suppressants.

## 4. Materials and Methods

### 4.1. Animals

Testes, pancreases, and aortas were collected from three-to-five-day old neonatal Duroc–Landrace pigs from the Texas Tech University New Deal Swine Unit. All animals were maintained in adherence to the approved Institute for Laboratory Animal Research Care, Use of Laboratory Animals, Texas Tech University Health Sciences Center Institutional Animal Care and Use Committee’s guidelines and protocols of the National Institutes of Health (protocol number 05019).

### 4.2. Pig Sertoli Cell, Islet, and Aortic Endothelial Cell Isolation

Testes were collected from four pigs from three different litters at three different farrowing times each, for a total of 12 pigs and 24 testes. Each litter of testes (n = 4 pigs, 8 testicles) were processed together for Sertoli cell isolation. Testes were sterilized for 30 s in 70% ethanol on ice two times, then stored in sterile Hank’s balanced salt solution (HBSS, Sigma-Aldrich, St. Louis, MO, USA) on ice during transit to the lab. NPSCs were isolated from pig testes with collagenase and trypsin digestion as described previously [2]. NPSCs were cultured as a single cell monolayer in tissue culture plates (Falcon, Corning, Inc., Corning, NJ, USA) as described below.

To isolate islets, pancreases were collected from the neonatal pigs and chopped into fragments (roughly 1–2 mm) in HBSS as previously described [38]. Tissue fragments were digested with type XI collagenase (sterile, 2.5 mg/mL, Sigma-Aldrich), then cultured for seven days at 37 °C and 5% CO_2_ in supplemented Ham’s F10 media with bovine serum albumin (BSA). Islets were cultured for at least a week to allow for purification of NPIs from acinar pancreatic cells [38], and then they were further cultured as described below.

Aortic endothelial cell isolation was performed as previously described [5]. Briefly, abdominal aortas were collected from the pigs and cut longitudinally to expose the inner lumen. Blood was removed through gentle irrigation with HBSS. To remove the endothelial cells, inner lumen was gently scraped with a cell scraper (Falcon, A Corning Brand, Corning, NY, USA). Cells were placed in a 50 mL conical tube and centrifuged three times at 1200 RPM for 10 min. Cells were plated in DMEM + 10% FBS at 37 °C, 5% CO_2_ and cultured as described below.

### 4.3. Human Complement Serum Cytotoxicity Assay

A HCS cytotoxicity assay was performed to measure cell viability after exposure to human anti-xenoantigen antibodies and human complement. The in vitro HCS cytotoxicity assays were performed similarly to that described previously [5]. Briefly, 200 K NPSC, 200 K NPIs, or 75 K pig aortic endothelial cells (PAECs) were plated per well on 24-well tissue culture plates (Becton Dickinson Labware, Franklin Lakes, NJ, USA) and cultured overnight in 1 mL DMEM media + 10% FBS (NPSC and PAECs). For islets, the tissue culture plates were coated with gelatin using EmbryoMax^®^ ultrapure water with 0.1% gelatin (Millipore, Burlington, MA, USA); then cells were plated and cultured overnight in 1 mL supplemented Ham’s F10 media with BSA. The next day, 500 μL of media was removed per well. Cells were treated in one of three groups: negative control group (addition of 500 μL serum-free media per well), complement group (addition of 500 μL pooled AB human complement serum per well, Innovative Research, Inc., Novi, MI, USA), and positive assay control group (addition of 500 μL 1% Triton X-100 detergent per well). Then cells were incubated at 37 °C for 90 min. Following incubation, media and treatments were removed from each well, and cell survival was assessed using the Cell Proliferation Kit I MTT (3-[4, 5-deimethylthiazol-2-yl]-2, 5-deiphenyltetrazolium bromide) assay (Millipore-Sigma, Darmstadt, Germany) per manufacturer’s instructions. Results were normalized to media-only wells. Three biological replicates were used.

### 4.4. NPSC-Conditioned Media in HCS Cytotoxicity Assay

Sertoli cell-conditioned media (SCCM) were made by culturing 15 × 10^6^ NPSCs (n = 5) in 150 mm tissue culture plates in exactly 35 mL of DMEM + 10% FBS. After 24 h incubation at 37 °C and 5% CO_2_, media were collected. SCCM was concentrated by placing the max volume of 20 mL of SCCM (or of DMEM + 10% FBS as a control) into Pierce™ Protein Concentrators (ThermoScientific, Waltham, MA, USA), which has a polyethersulfone filter with a molecular weight cut off at 10 K. Protein concentrator with media were centrifuged for 15 min at 5000× *g* per manufacturer’s instructions and concentrated SCCM was stored at −80 °C until used. Roughly 12 mL of SCCM was obtained through this method. The HCS cytotoxicity assay was performed as previously described with the following conditions. Seventy-five thousand PAECs were plated per well on a 24-well tissue culture plate and cells were cultured overnight. All media were removed and 500 μL of either fresh DMEM + 10% FBS, concentrated DMEM + 10% FBS, or concentrated SCCM was added gently to each well. The rest of the assay was performed as described above.

### 4.5. RNA Sequencing

To prepare NPSCs for RNA sequencing, three million NPSCs were cultured on 150 mm tissue culture plates (Corning, Inc., Corning, NJ, USA) in 10 mL DMEM + 10% FBS or overnight at 37 °C and 5% CO_2_. The next day, media were removed, and cells were washed with phosphate buffered saline (PBS, pH 7.4). Cells were carefully scraped off the plates and lysed with 1 mL of Trizol^®^ reagent (Ambion by Life Technologies, Carlsbad, CA, USA). Samples were stored at −80 °C until all samples were collected (n = 3). Once all samples were collected, they were packaged in dry ice and shipped to GENEWIZ from Azenta Life Sciences (South Plainfield, NJ, USA) for RNA isolation, RNA sequencing, and data processing. RNA sequencing data on NPIs were previously performed by Kim et. al. [39] and are publicly available in the National Center for Biotechnology Information (NCBI) Gene Expression Omnibus (GEO, accession number GSE143889) [40]. RNA sequencing data on PAECs were previously performed by Wang et. al. [41] and are publicly available in the NCBI GEO (GSE196055); however, the PAEC dataset only contained averaged expression and not raw data on each n. RNA sequencing data on human testicular tissue were previously performed by Guo et. al. [32] and are publicly available in the NCBI GEO (GSE120508). All numerical RNA sequencing data are in transcripts per million (TPM).

### 4.6. RNA Isolation and PCR of Complement Inhibitors

To isolate total RNA for quantitative RT-PCR (qPCR) experiments, the Purelink^TM^ RNA Mini Kit (Invitrogen, Carlsbad, CA) was used, and the manufacturer’s protocol was followed. Briefly, cells were suspended in 0.6 mL lysis buffer with 1% 2-mercaptoethanol (BioRad Laboratories, Hercules, CA, USA), then passed 10 times through a sterile 21-gage needle. RNA was isolated through a series of washes and filters, then stored at −80 °C until ready for cDNA conversion and qPCR. RNA was quantified using a nanodrop (Nanodrop one, Thermo Fisher Scientific, Waltham, MA, USA) at 260 nm. Total RNA was reverse transcribed into cDNA using the High-Capacity cDNA Reverse Transcriptase Kit with RNase Inhibitor (Applied Biosciences, Waltham, MA, USA) and the ProFlex PCR System thermocycler (Applied Biosciences, Waltham, MA, USA).

qPCR was performed on cDNA samples for target complement genes (Table 3) with QuantStudio^TM^ 3 Real Time PCR system (Applied Biosystems by Thermo Fischer Scientific, Waltham, MA, USA) with iTaq Universal SYBR^®^ Green Supermix (Bio Rad Laboratories, Inc., Hercules, CA, USA). All target gene expression was normalized to a GAPDH endogenous control. Gene expression was calculated through the following formula:

Gene expression = 2 − (ΔCT × 1000) = 1,(1)

Forward and reverse primers for the target genes C1INH, CD35, CD46, CD55, CFH, CLU, COMP, CPN2, CSMD1, and PTX3 are contained in Table 3.

### 4.7. Protein Isolation and Quantification with ELISA

Total cellular proteins (n ≥ 3) were isolated from NPSCs and PAECs using lysis with RIPA buffer (20 mM Tris-HCl (pH 7.5), 150 mM NaCl, 1% NP-40, 0.1% SDS, 1% deoxycholic acid, 5 mM NaF, 1 mM EDTA) containing PMSF and protease inhibitor cocktail (Sigma) for 30 min on ice followed by centrifugation at 12,000× *g* for 5 min. Protein concentrations were determined using the Bradford assay (BioRad, Hercules, CA) with BSA as the standard [42].

ELISA assays were used to quantify secreted protein levels of Cpb2 and Ptx3 (MyBioSource, Inc., San Diego, CA, USA) in NPSC- and NPI-conditioned media. ELISA assays were performed per the manufacturer’s protocol. In short, provided standards or diluted samples (1:4 dilution of non-concentrated CM) were added into detection antibody pre-coated wells, followed by biotinylated detector antibody, avidin-horse radish peroxidase, 3,3′,5,5′-tetramethylbenzidine (TMB) substrate, then stop solution. After addition of stop solution, plates were read immediately at 450 nm O.D. absorbance.

### 4.8. Statistical Analysis

All values are expressed as means ± standard error of mean and were compared using one-way ANOVA or unpaired t-test per row and individual variances computed for each comparison. Statistical significance between groups was set at *p* < 0.05. All statistical analyses were performed using GraphPad Prism9 software (Dotmatics, San Diego, CA, USA). Percentages of similarity between RNA sequencing and qPCR results were calculated per each gene. To evaluate concordance in gene expression intensities between RNA-sequencing and qPCR, we first calculated expression correlation between normalized RT-qPCR C_q_-values and log transformed RNA-seq expression values.

## 5. Conclusions

The complement system has historically been understudied but is presently shown to be an important modulator of total immune function. We now know that complement plays many critical roles in fighting infections, neuronal development, fertility, immune cell differentiation, and immune function. Regarding transplant rejection, complement has been shown to cause excessive collateral damage to the patient, so complement inhibition and regulation is relevant in understanding graft survival. Ergo, the identification of the Sertoli cell complement signature is significant since it allows us to better elucidate what proper complement regulation can do in a graft survival environment. As we better understand this, we will be able to develop more effective and less toxic therapies for transplant recipients. This is especially timely with the current exciting clinical xenograft studies.

## Figures and Tables

**Table 1:** Functions of complement inhibitors expressed by NPSCs.

Inhibitor	Mass (kD)	Distribution	Target	Function in Complement
C1INH	85–105	Plasma	C1r, C1s	Dissociates C1r/s from C1q
C4BP	540–590	Plasma	C4b	Prevents assembly of CP C3 convertase
CD35	190–250	Membrane	C1q, MBL, C4b, C3b	Competitively binds C1q/MBL/C4b/C3b
CD46	51–68	Membrane	C4b, C3b	Cleaves C3b/C4b
CD55	60–75	Membrane	C3b	Decays convertases, inhibits C3 cleavage
CD59	18–23	Membrane	C8	Suicide inhibition binding of C8
CFH	115	Plasma	C3b, Bb	Cleaves C3b, dissociates Bb from C3b
CFI *	88	Plasma	C4b, C3b	Inactivates C4b/C3b by cleaving α chains
CLU	34–80	Both	C6, C7, C8, C9	Inhibits MAC assembly, prevents C9 insertion
COMP	524	Plasma	C1q, MBL, C3	Prevents C1q/MBL binding, stabilizes C3
CPB2/N1/N2	47–60	Plasma	C3a, C5a	Removes carboxy terminus to form desArg
CSMD1	389	Membrane	C3b, C7	Cleaves C3b, prevents C7 binding to C6
PLG	92	Both	C3, C3b, C3d, C5	Decays convertases, inhibits C3
PTX3	175	Plasma	C1q	Prevents C1q activation/binding
SMAP1/2	94	Plasma	MASP1-3	Binds MASP1-3 and prevents their action
SUSD4	49	Membrane	C1q, MBL	Prevents C1q/MBL binding and C2 cleavage
VTN	75	Plasma	C7	Binds C7 to prevent C8 recruitment
VWF	500–20,000	Plasma	C3b	Cleaves C3b

* Requires one of the following cofactors: C4BPA/B, CSMD1, CFH, CD35, CD46. Table references [6,7,8,9,10].

**Table 2:** Cellular expression of complement inhibitors, factors, and receptors.

Cell Type	Complement Inhibitors	Complement Factors	Complement Receptors
**Sertoli cells (pig)**	C1INH, C4BP, CD35, CD46, CD55, CD59, CFH, CFI, CLU, COMP, CPB2, CPN1, CPN2, CSMD1, PLG, PTX3, SMAP1, SMAP2, SUSD4, VTN, VWF	C1q, C1s, C2, C3, C4, C5, C6, C7, C8, C9, CFB, CFD, CFP, FCN1, FCN2, MASP1, MASP2, MBL1, MBL2	CD35, C5aR1, C5aR2
**Testicular tissue cells (human)**	C1INH, CD35, CD46, CD55, CD59, CRIg, CFH, CFHR1, CFI, CLU, COMP, CPB1, CPN1, CSMD1, PTX3, SMAP1, SMAP2, SUSD4, VTN, VWF	C1q, C1s, C2, C3, C4, C5, C6, C7, C8, C9, CFD, CFP, FCN1, MASP1	CD21, CD35, C3aR, C5aR1, C5aR2, CRIg
**Complement-producing cells**			
**Hepatocytes**	C1INH, C4BP, CD46, CD55, CD59, CFH, CFHR1-5, CFI, CLU, PLG, PTX3, SMAP1, SMAP2, VTN	C1r, C1s, C2, C3, C4, C5, C6, C8, C9, CFB, FCN1, FCN2, FCN3, MBL, MASP1, MASP2, MASP3	C5aR2
**Renal cells**	CD35, CD46, CD55, CD59, CLU, PLG, PTX3, SMAP1, SMAP2, VTN, VWF	C1q, C2, C3, C4, CFB, CFD, C9	CD35
**Immune cells**			
**Neutrophils**	CD35, C4BP, CD46, CD55, CD59, CLU, PTX3, SMAP1, SMAP2	C3, C6, C7, CFB, CFP, FCN1	CD35, CR3, CR4, C3aR, C5aR1
**Monocytes**	CD35, C1INH, C4BP, CFH, CFI, CLU, PTX3, SMAP1, SMAP2	C1q, C1r, C1s, C2, C3, C4, C5, C6, C7, C8, C9, CFB, CFD, CFP	CD35, CR3, CR4, C3aR, C5aR1
**Macrophages**	C1INH, CD35, CFH, CFI, CLU, PTX3, SMAP1, SMAP2	C1q, C1r, C1s, C2, C3, C4, C5, CFB, CFD, CFP	CD35, CR3, CR4, CRIg, C3aR, C5aR1, C5aR2
**Dendritic cells**	C4BP, CD35, CFH, CFI, CLU, PTX3, SMAP1, SMAP2	C1q, C1r, C1s, C2, C3, C4, C5, C7, C8, C9, CFB, CFD, CFP	CD35, CR3, CR4, CRIg, C3aR, C5aR1
**B cells**	CD35, CD46, CD55, CD59, CLU, PTX3, SMAP1, SMAP2	C5	CD35, CD21, CR4, C5aR1
**T cells**	C1QBP, CD35, CD46, CD55, CD59, CFH, CLU, PTX3, SMAP1, SMAP2, SUSD4	C3, C5, CFB, CFD, CFP	C1qBP, CD35, C3aR, C5aR1, C5aR2

Information on all cells except for Sertoli cells is human. NK: natural killer. CRIg: complement receptor of the immunoglobulin superfamily. Human Sertoli cell gene expression data from Guo et. al., 2018 [32]. Chart references [16,23,24,25,33,34,35,36].

**Table 3:** Pig PCR primer sequences designed using NCBI PrimerBlast.

Gene	Forward Primer (5′ → 3′)	Reverse Primer (5′ → 3′)
*C1INH ^1^*	GAC CAA GTT CTA TCC CAC TCA C	CAG GTT GAG GTC GTA GGT AAA G
*CD35*	GAG TTT GGA GCA GCC TTC CT	GGC ACC CAT GTG TTG TTG AC
*CD46*	ATC GCT GCA ATT GTT GTG GG	GAT TCC ACG TCC TCT CAG CA
*CD55*	GAC TTG TTA TTT GGC GCA TCC	ATC GCC AGT CGC AGG TAA AT
*CFH*	GAC ACG ACC TCA TTC CCA TTA C	GTT CTG ACC ACY GTC CAT TCT C
*CLU*	GCA GAA TGA CGA CCG CTA CT	CGT GAG ATT CAC CTC TCA CTC C
*COMP*	GGA CAG TGA TGG TGA TGG TAT AG	TCA CAT GCA TCT CCC ACA AA
*CPN2*	TGC TTC ATC CAC GAG GTG TT	ACC ACT TTG GTC AGG TTG GG
*CSMD1*	GGC TTT GTG GAA AAT GCC GT	TGC ACG AGT AGT GAA CCA CC
*GAPDH ^2^*	CCT CCC CGT TCG ACA GAC A	GAT GCG GCC AAA TCC GTT
*PTX3*	AGC AGG TTG TGA AAC AGC GA	GTT TCA TTG GTG TTG CCG GG

^1^ Common gene name is *SERPING1*. ^2^ Endogenous control.

## Data Availability

The data presented in this study are openly available in the NCBI GEO database, accession number GSE221711.

## Supplementary Materials

The following supporting information can be downloaded at https://www.mdpi.com/article/10.3390/ijms24031890/s1.
