# Supplementary material for: The Sertoli Cell Complement Signature: A Suspected Mechanism in Xenograft Survival
Source: Int J Mol Sci. 2023 Jan 18;24(3):1890. doi: 10.3390/ijms24031890 (PMC9916409; doi:10.3390/ijms24031890)
Supplement: Supplementary file 1 [file ijms-24-01890-s001.zip › ijms-2161806-supplementary.pdf]

Table S1. Expression of complement factors by NPSC and NPI as determined by RNA sequencing.

Gene	NPSC	NPI	FC	LFC	P-value
C1QA	11.06	9.96	0.10	3.33	0.836346
C1QL2	6.83	0.01	1.00	0.00	0.002023
C1QL3	6.41	0.04	0.99	0.01	0.001497
C1QL4	6.53	0.16	0.98	0.03	0.026016
C1R	0.00	8.95	-8945.56	-13.13	0.014513
C2	9.60	1.16	0.88	0.18	0.04208
C3	86.65	79.59	0.08	3.62	0.773536
C4B	66.54	2.74	0.96	0.06	0.016524
C5	1.90	2.80	-0.48	-1.06	0.019483
C5AR1	8.23	2.14	0.74	0.44	0.005229
C5AR2	9.22	0.04	1.00	0.01	0.001192
C6	1.58	24.85	-14.73	-3.88	0.00766
C7	6.79	1.82	0.73	0.45	0.064896
C8A	2.83	0.17	0.94	0.09	0.004586
C8B	3.69	4.44	-0.20	-2.32	0.44578
C8G	5.79	0.46	0.92	0.12	0.108388
C9	6.20	0.00	1.00	0.00	0.000603
CFB	11.90	2.62	0.78	0.36	0.015629
CFD	19.39	64.60	-2.33	-1.22	0.128008
CFP	9.70	35.35	-2.65	-1.41	0.094532
FCN1	9.73	0.45	0.95	0.07	0.211552
FCN2	10.30	0.00	1.00	0.00	0.171437
MASP1	4.27	0.27	0.94	0.10	0.014418
MASP2	14.53	2.01	0.86	0.21	0.008284
MBL1	3.61	4.66	-0.29	1.79	0.542053
MBL2	2.31	0.00	1.00	0.00	0.021469

Values are expressed as mean transcripts per million. Data in this table corresponds to the charts in Figure 8. FC: fold change. LFC: Log2 Fold Change.

Table S2. Summary of complement inhibitor expression by NPSC and NPI as determined by RNA sequencing.

Gene	NPSC	NPI	FC	LFC	p-value
C1INH	109.80	19.91	0.82	0.29	0.001436
C4BP	2.88	39.46	-12.72	-3.67	0.160961
CD35	7.40	0.00	1.00	0.00	0.007392
CD46	1.23	0.99	0.20	2.32	0.607071
CD55	36.89	9.34	0.75	0.42	0.000068
CD59	15.93	15.35	0.04	4.78	0.495616
CFH	78.35	29.49	0.62	0.68	0.001117
CFI	4.56	9.41	-1.07	-0.10	0.087802
CLU	1923.00	602.60	0.69	0.54	0.013758
COMP	23.96	0.00	1.00	0.00	0.004941
CPB2	17.07	33.05	-0.94	-0.09	0.22039
CPN1	34.10	13.82	0.59	0.75	0.061457
CPN2	23.78	0.24	0.99	0.01	0.022721
CSMD1	3.79	0.08	0.98	0.03	0.018506
PLG	3.66	1.77	0.52	0.95	0.12442
PTX3	8.73	0.35	0.96	0.06	0.003628
SMAP1	12.76	66.55	-4.22	-2.08	0.124607
SMAP2	19.26	49.47	-1.57	-0.65	0.055024
SUSD4	27.05	33.57	-0.24	-2.06	0.530789
VTN	1.06	3.65	-2.43	-1.28	0.110116
VWF	47.38	25.98	0.45	1.15	0.134216

Values are expressed as mean transcripts per million. Data in this table corresponds to the chart in Figure 3A. FC: fold change. LFC: Log2 Fold Change.

Table S3. Expression of complement inhibitors by NPSC and PAEC as determined by RNA sequencing.

Inhibitor	NPSC	PAEC	FC	LFC
C1INH	109.80	8.73	0.92	0.12
C4BP	2.87	0.48	0.83	-3.67
CD35	7.40	0.02	1.00	0.00
CD46	1.23	31.93	-24.90	-4.64
CD55	36.89	0.35	0.99	0.01
CD59	15.93	0.11	0.99	0.01
CFH	78.35	0.38	1.00	0.01
CFI	4.56	0.35	0.92	-0.10
CLU	1923.00	6.96	1.00	0.01
COMP	23.96	0.02	1.00	0.00
CPB2	17.07	0.01	1.00	0.09
CPN1	34.10	0.01	1.00	0.00
CPN2	23.78	0.07	1.00	0.00
CSMD1	3.79	0.04	0.99	0.02
PLG	3.66	0.01	1.00	0.01
PTX3	8.73	0.00	1.00	0.00
SMAP1	12.76	3.14	0.75	0.41
SMAP2	19.29	25.63	-0.33	-1.60
SUSD4	27.05	0.15	0.99	2.06
VTN	1.06	0.77	0.27	1.28
VWF	47.38	0.03	1.00	0.00

Values are expressed as mean transcripts per million. Data in this table corresponds to the chart in Figure 3B. FC: fold change. LFC: Log2 Fold Change.

Table S4. Raw CT values for qPCR of complement inhibitors.

Gene	NPSC	NPI	PAEC
**C1INH**	20.74 ± 0.24	23.86 ± 0.43	25.55 ± 0.35
**CD35**	23.02 ± 0.37	30.46 ± 1.92	NA
**CD55**	26.84 ± 0.05	31.21 ± 0.21	NA
**CFH**	22.01 ± 0.39	22.25 ± 0.30	29.68 ± 0.40
**CLU**	17.40 ± 0.38	17.99 ± 0.32	19.52 ± 0.49
**COMP**	26.05 ± 0.15	29.02 ± 0.23	30.44 ± 1.01
**CPN2**	24.88 ± 0.03	28.53 ± 0.88	29.73 ± 0.50
**CSMD1**	27.73 ± 0.31	29.51 ± 0.30	NA
**GAPDH**	18.76 ± 0.11	18.79 ± 0.05	17.09 ± 0.05
**PTX3**	23.51 ± 0.88	28.39 ± 0.81	29.12 ± 1.02
**SUSD4**	27.24 ± 0.33	NA	30.97 ± 0.55

Data are expressed as mean cycle threshold (CT) ± standard error. The lower the CT value, the higher the gene is expressed. NA: not analyzed for this cell set.

Table S5. Summary of complement inhibitor expression by NPSC and NPI as determined by qPCR.

Gene	NPSC	NPI	FC	LFC	p-value
C1INH	1.00	0.14	0.86	0.22	0.000685
CD35	1.01	0.03	0.97	0.04	0.000209
CD46	1.00	1.12	-0.12	-3.06	0.003034
CD55	1.01	0.16	0.85	0.24	0.001256
CFH	1.00	1.19	-0.19	-1.66	0.239762
CLU	1.01	0.69	0.32	1.66	0.000003
COMP	1.01	0.13	0.87	0.20	<0.000001
CPN2	1.00	0.09	0.91	0.13	0.012508
CSMD1	1.01	0.35	0.65	0.62	0.002953

NPSC and NPI data are expressed as mean fold difference. Data in this table corresponds to the chart in Figure 5A. FC: fold change. LFC: Log2 Fold Change.

Table S6. Summary of complement inhibitor expression by NPSC and PAEC as determined by qPCR.

Gene	NPSC	PAEC	FC	LFC	p-value
C1INH	1.01	0.01	0.99	0.02	<0.000001
CFH	1.01	0.00	1.00	0.00	0.000002
CLU	1.00	0.04	0.12	3.06	0.000019
COMP	1.00	0.01	0.99	0.01	<0.000001
CPN2	1.01	0.02	0.19	2.40	0.000001
CSMD1	1.01	0.02	0.98	0.04	0.000002
PTX3	1.01	0.05	0.95	0.07	<0.000001
SUSD4	1.01	0.01	0.99	0.02	<0.000001

NPSC and PAEC data are expressed as mean fold difference. Data in this table corresponds to the chart in Figure 5B. FC: fold change. LFC: Log2 Fold Change.
